# Cardiomyopathy Management and In-Hospital Outcomes in a Tertiary Care Center: Clinical Components and Venues of Advanced Care

**DOI:** 10.7759/cureus.19054

**Published:** 2021-10-26

**Authors:** Sheeren Khaled, Emad M Babateen, Faisal Y Alhodian, Renad W AlQashqari, Reema S AlZaidi, Hala Almaimani, Nadin A Alharbi, Kawlah E Samarin, Amani A Fallatah, Ghada Shalaby

**Affiliations:** 1 Cardiology, Cardiac Center, King Abdullah Medical City, Makkah, SAU; 2 Cardiology, Faculty of Medicine, Benha University, Benha, EGY; 3 Cardiology, College of Medicine, King Saud Bin Abdulaziz University for Health Sciences, King Abdullah International Medical Research Centre, King Abdulaziz Medical City, National Guard Health Affairs, Jeddah, SAU; 4 Surgery and Medicine, King Abdulaziz University, Jeddah, SAU; 5 Cardiology, Ibn Sina National College for Medical Studies, Jeddah, SAU; 6 Cardiology, Medicine and Surgery, Taif University, Taif, SAU; 7 Cardiology, College of Medicine, Umm Alqura University, Makkah, SAU; 8 Cardiology, King Abdulaziz University, Jeddah, SAU; 9 Cardiology, Faculty of Medicine, Zagazig University, Zagazig, EGY

**Keywords:** in-hospital outcomes, advanced care, management, clinical features, cardiomyopathy

## Abstract

Background

There are few reports on the prevalence of different types of cardiomyopathy, clinical presentation, severity, short-term outcomes, and implementation of advanced heart failure treatment. This study aimed to assess the prevalence, clinical background of different types of cardiomyopathy and to identify the candidate for advanced treatment in a tertiary care cardiac center with many advantages

Method

A single-center retrospective cohort study included 1069 patients admitted to our center and diagnosed with cardiomyopathy during 2019 and 2020

Results

Out of 1069 cardiomyopathy patients admitted and diagnosed at our center between 2019 and 2020, 62% had ischemic cardiomyopathy (ICM), 36% had dilated cardiomyopathy (DCM), and 2% had hypertrophic cardiomyopathy (HOCM). ICM patients were older, showed a higher prevalence of both male gender and pilgrims, and they had more frequent cardiovascular risk factors compared to dilated cardiomyopathy group of patients. However, DCM patients with more severe heart failure symptoms (NYHA class III/IV), much worse LVEF, were subsequently considered deemed for aggressive diuretic therapy, and further advanced therapy (Sacubitril-Valsartan and device therapy) compared to ICM patients. ICM patients showed poor in-hospital outcomes compared to DCM group of patients (0.05 and <0.001) for an indication for mechanical ventilation and in-hospital mortality, respectively). Increased age, presence of renal dysfunction and lower LVEF were found the independent predictors of in-hospital mortality among our studied patients

Conclusion

There are discrepancies between DCM and ICM patients. Although DCM patients were younger at age and had fewer cardiovascular risk factors, they presented with severe symptoms and dysfunction, hence more eligible candidates for advanced heart failure treatment, and finally showed a lower mortality rate. Increased age, presence of renal dysfunction and lower LVEF were found the independent predictors of in-hospital mortality.

## Introduction

Cardiomyopathies are a group of disorders affecting the heart muscle leading to problems in the function and structure of the myocardium [1]. It is defined as a heterogeneous group of diseases of the myocardium associated with mechanical and/or electrical dysfunction, which usually (but not invariably) exhibit inappropriate ventricular hypertrophy or dilatation, due to a variety of etiologies that are frequently genetic [2]. Dilated cardiomyopathy is characterized by left ventricular (LV) dilatation and dysfunctional contractility [3], while ischemic cardiomyopathy is caused by a defect in the myocardial perfusion leading to ischemic manifestations [4]. Hypertrophic cardiomyopathy is an autosomal dominant disorder caused by a missense genetic mutation and results from asymmetric septal hypertrophy causing outflow obstruction of the left ventricle [2,3]. 

With the continuing advancement of cardiomyopathy management; Sacubitril/Valsartan (Entresto), which is a combination of Sacubitril (a neprilysin inhibitor) and Valsartan (an angiotensin receptor blocker) has been recently introduced as a medical therapy [5]. Another strategy is device therapy, which includes cardiac resynchronization therapy (CRT) and implantable cardioverter-defibrillators (ICDs) [6].

Little is known about the clinical presentation, severity, short-term outcomes, and implementation of advanced heart failure treatment among cardiomyopathy patients in the Middle Eastern region. King Abdullah Medical City (KAMC) is the only center in the Mecca region providing tertiary care facilities such as revascularization and advanced heart failure treatment. Because of this, it receives most of the cardiomyopathy patients deemed suitable for further workup, including invasive assessment and advanced management. This made us a unique institution to conduct such a study. 

This study aimed to review the prevalence and clinical background of different types of cardiomyopathies with the identification of the candidates for advanced treatment in a tertiary care cardiac center and assessing the effect of this treatment on short-term outcomes. 

The abstract of this study was presented orally in Cardio Alex 1-4 June 21. Also, it was submitted to ESC congress 2021.

## Materials and methods

Study population

This was a single-center retrospective cohort study in which the data was retrospectively collected from hospital records in KAMC, a tertiary care hospital in Mecca, Saudi Arabia. The study included 1069 patients who were admitted to our cardiac center (either directly from the emergency department or referral cases from other hospitals) and diagnosed with cardiomyopathy during 2019 and 2020. 

Ethical approval

This study was approved by the hospital’s institutional review board (IRB number 20-660). 

Inclusion criteria

Patients were diagnosed based on symptoms of clinical presentation (chest pain and/or heart failure symptoms) and imaging (echocardiographic and coronary angiography) data. 

Echocardiography and diagnostic standard criteria 

All standard echocardiography parameters were collected: LVEF, LV size, left atrium (LA) size, right ventricle (RV) size and function, assessments of valves including mitral regurgitation (MR), and left ventricular apex for left ventricular thrombus.

- For LVEF, Biplane Method of Disks (modified Simpson’s rule; LVEF = LVEDV - LVESV / LVEDV) was used for calculation [7].

- For LV size, Biplane Method of Disks (modified Simpson’s rule) is used for chamber quantification (Severe LV dilatation was defined as LV diastolic volume/BSA (Body surface area) of > 100mL/m^2^ in men and > 80mL/m^2^ in women , and LV systolic volume/ BSA of > 45 mL/m^2^ in men and > 40mL/m^2^ in women) [7].

- For LA size, volume is calculated using modified biplane method (Severe LA dilation was defined as LA volume/ BSA > 48 mL/m^2^) [7].

- For RV size, multiple acoustic windows for chamber quantification were used with inner-edge to inner-edge measurements ( RV dilation was defined as > 41, 35, 83, 30, 35 and 27 at RV basal, mid, longitudinal dimensions, RVOT PLAX (RV outflow tract at parasternal long-axis view), RVOT proximal and distal diameters, respectively) [7]. Severe RV dilation was defined as RV/LV volume ratio ≥2.30.

- For RV function, TAPSE (Tricuspid Annular Plane Systolic Excursion) and DTI-Derived Tricuspid Lateral Annular Systolic Velocity S’ were used for assessment (RV dysfunction was defined as TAPSE <17 mm and/or Pulsed Doppler S' <9.5 cm/sec) [7]. Moderate-severe RV dysfunction was defined as TAPSE <15 mm.

- For Mitral Regurgitation (MR), significant MR was defined as grade III/IV (Effective Regurgitant Orifice Area (EROA)> 0.30 mm^2^, Regurgitant Volume (RVol)> 45 mL and Rgurgitant Fraction (RF)> 40%).

- A special zoom on the left ventricular (LV) apex was applied and harmonic imaging was used because the majority of thrombi were located at the apex

I- For DCM: Left ventricular ejection fraction (LVEF) <0.40 (>2SD) and/or factional shortening <0.25 (> 2 SD), as well as a left ventricular end-diastolic diameter > 117% of the estimated value corrected for age and body surface according to Henry equation: (45·3(BSA) 1/3"0·03(age)"7·2) which corresponds to 2 SD of the predicted normal limit +5%) [8]. 

II- For ICM: Diagnostic criteria were similar to DCM in addition to coronary artery disease obstruction ( ≥50% narrowing of the diameter of the lumen of the left main coronary artery or ≥70% narrowing of the diameter of the lumen of the left anterior descending coronary artery, left circumflex artery or right coronary artery). 

III- for HCM: In the absence of secondary causes of hypertrophy (HTN, Aortic stenosis), it is diagnosed based on ≥ 15 mm wall thickness in one or more myocardial segments measured by echocardiography [9]. 

Exclusion criteria 

All patients out of the scope of service and <18 years old were excluded. 

All patients admitted before 2019 were excluded due to incomplete data. 

All patients with other types of cardiomyopathy (restrictive, LV non-compaction, stress-induced cardiomyopathy, etc.) were excluded due to a very small number with incomplete data.

Patients with HCM were encountered in small numbers with different characteristics and were not addressed in the current study.

Clinical and hospital course data collected for all patients included the following 

Demographic data: age, gender, body mass index (BMI), and status (residence/pilgrims). 

Risk factors: diabetes mellitus (DM) [10], hypertension (HTN) [10], smoking, dyslipidemia (DLP) [10], presence of chronic kidney disease (CKD), old cerebrovascular accidents (CVA), and history of chronic obstructive pulmonary disease (COPD). 

Clinical presentation and laboratory results: severe heart failure symptoms (New York Heart Association (NYHA) functional classification III/IV). Blood urea, serum creatinine, sodium, potassium, and brain natriuretic peptide (BNP) markers were all monitored. 

Standard medications: beta-blockers (BB), spironolactone, angiotensin-converting enzymes inhibitors (ACEIs)/angiotensin-receptor blockers (ARBs), loop diuretics (Lasix), metolazone, digoxin. 

Advanced treatment strategies including the use of advanced heart failure treatment and revascularization for ICM: advanced heart failure therapy was planned for selected patients who were fulfilled the following criteria- 

Sacubitril/Valsartan (Entresto) for patients with NYHA Class II-IV and reduced ejection fraction without drug contraindications or limitations [5]. 

Device treatment using ICDs for primary or secondary prevention of sudden cardiac death and CRT-D for patients who remain in NYHA functional classes II and III despite optimal medical therapy with a wide QRS complex and reduced left ventricular ejection fraction (LVEF ≤30% to 35%) [6]. 

Revascularization therapy for ICM patients includes percutaneous coronary intervention (PCI) and coronary artery bypass grafting (CABG). 

Hospital outcomes: in-hospital death, length of stay (LOS), left ventricular ejection fraction (LVEF) [7], pulmonary edema, cardiac arrest, cardiogenic shock, history of mechanical ventilation (MV), and history of left ventricular thrombus (LVT). 

Statistical analysis

Statistical analysis was performed using the SPSS software package (SPSS Inc.; Chicago, IL) version 21.0. A descriptive statistical analysis was carried out by reporting the number and percentage for categorical variables and the mean and standard deviation for continuous variables. Demographic and clinical data, as well as close-ended questions, were summarized in frequency tables. In the comparison between DCM and ICM groups, a chi-squared test was used for categorical variables, while a t-test was used for continuous variables. For all analyses, a P-value of < 0.05 was considered significant and a value of > 0.05 was not considered significant. For the multivariate analyses, we performed Poisson regression with an estimation of robust variances using stepwise methodology to calculate the incidence ratios and the 95% confidence intervals 

## Results

**Figure 1:**
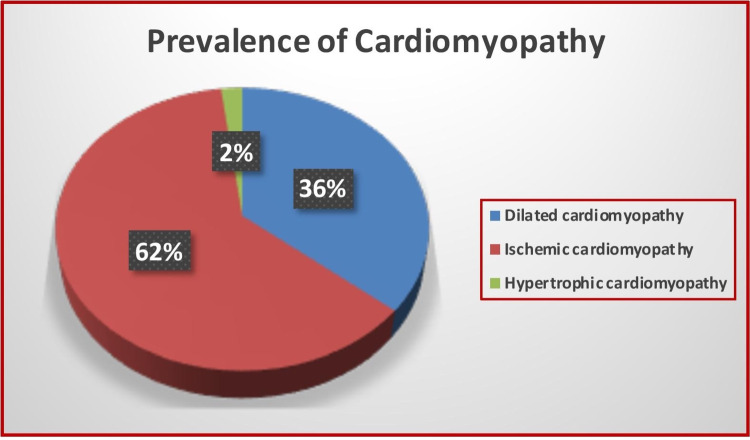
Prevalence of different types of cardiomyopathy.

All patients underwent admission and investigation and were diagnosed with cardiomyopathy during their hospital stay in 2019 and 2020. They were divided into three groups: (1) dilated cardiomyopathy (DCM): 385 patients (36% of 1069); (2) ischemic cardiomyopathy (ICM): 663 patients (62% of 1069); and (3) hypertrophic cardiomyopathy (HOCM): 21 patients (2% of 1069), as shown in Figure 1.

We compared patients with reduced left ventricular ejection fraction (LVEF <40%) with DCM versus ICM in terms of baseline clinical data, treatment, and in-hospital outcome measures. 

Clinical data 

As shown in Table 1, ICM patients were of an older age, showed higher a prevalence of both male gender and pilgrim status, and had more frequent cardiovascular risk factors when compared to DCM patients (P<0.001, 0.001, and 0.004 for DM, HTN, and CKD, respectively). 

**Table 1:** Baseline demographic and clinical data of dilated and ischemic cardiomyopathy patients. BMI: body mass index; BNP: brain natriuretic peptide; CKD: chronic kidney disease; COPD: chronic obstructive lung disease; CVA: cerebrovascular accidents; DLP: dyslipidemia; DM: diabetes mellitus; HTN: hypertension; NS: not significant.

Variable	Dilated cardiomyopathy (DCM), N = 385 (36%)	Ischemic cardiomyopathy (ICM), N = 663 (62%)	P-value
Age (years)	50.39± 13.3	59.43± 11.6	<0.001
Male gender	265 (69%)	583 (88%)	<0.001
Pilgrims	4 (1%)	86 (13%)	<0.001
BMI (kg/m^2^)	30.1±6.9	28.7±6.1	0.017
DM	181 (47%)	477 (72%)	<0.001
HTN	219 (57%)	471 (71%)	0.001
DLP	100 (26%)	179 (27%)	NS
Smoking	123(32%)	240 (36%)	NS
Family history of cardiomyopathy	21 (5.5%)	22 (3.3%)	NS
CKD	42 (11%)	141 (21%)	0.004
H/O CVA	54 (14%)	140 (21%)	NS
H/O COPD	23 (6%)	41 (6%)	NS
Serum urea (mg/dl)	55.32± 36.7	54.31± 34.8	NS
Serum creatinine (mg/dl)	1.1± 1.9	1.31± 1.5	0.04
Serum sodium (mg/dl)	137.35± 4.1	136.25± 3.2	<0.001
Serum potassium (mg/dl)	4.23± 0.4	4.23± 0.3	NS
BNP on admission (pg/ml)	1159.09± 2529.3	919.76± 1202.5	0.07

No significant difference was found between the two groups regarding associated morbidities in the form of a history of CVA or COPD. ICM patients showed more hyponatremia (P<0.001) and higher serum creatinine (P=0.04) compared to DCM patients. Higher values of BNP were detected among DCM patients. 

**Figure 2:**
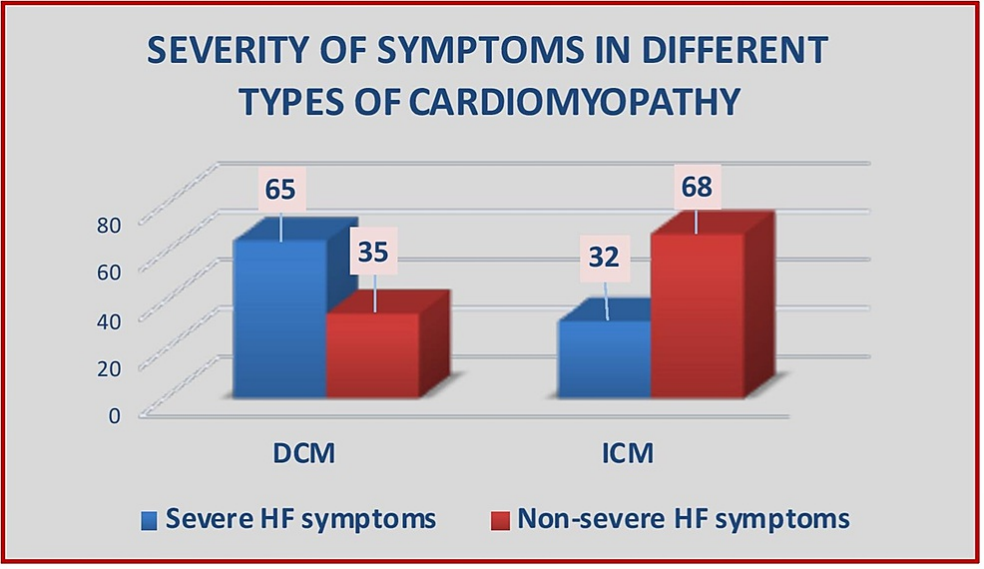
Severe heart failure symptom presentation in different types of cardiomyopathy. DCM: dilated cardiomyopathy; ICM: ischemic cardiomyopathy; HF: heart failure.

More than half of patients with DCM (431, 65%) presented with severe heart failure symptoms (NYHA class III/IV) and needed intensive anti-failure treatment; however, only a third (123, 32%) of ICM patients group had severe symptoms (P<0.001), as shown in Figure 2.

The majority of ICM patients presented with chest pain and acute coronary syndrome (32% presented with ST-elevation myocardial infarction, 25% with non-ST-elevation myocardial infarction, and only 11% with unstable angina). 

Table 2 shows that, when examined using echocardiography, patients with DCM had more deteriorated function (severe LA, LV, and RV dilatation [P<0.001, <0.001, and 0.005, respectively]), significant MR (P=0.02), and lower LVEF (P<0.001) compared to patients with ICM. No significant difference in RV systolic dysfunction nor left ventricular thrombus was noted between the two groups. 

**Table 2:** Echocardiographic findings of dilated and ischemic cardiomyopathy patients. LA: left atrium; LV: left ventricle; LVEF: left ventricular ejection fraction; LVT: left ventricular thrombus; MR: mitral regurgitation; RV: right ventricle; NS: not significant.

Variable	Dilated cardiomyopathy (DCM); N = 385 (36%)	Ischemic cardiomyopathy (ICM); N =663 (62%)	P-value
Severe LV dilatation	135 (35%)	80 (12%)	<0.001
Severe LA dilatation	89 (23%)	47 (7%)	<0.001
Severe RV dilatation	27 (7%)	7 (1%)	0.005
Significant MR (III/IV)	150 (39%)	199 (30%)	0.02
LVEF%	25.8 ±9.4	39.1± 9.1	<0.001
Significant RV dysfunction	70 (18%)	93 (14%)	NS
LVT	35 (9%)	67 (10%)	NS

Management strategies 

Aggressive diuretic treatment was used more often among DCM patients than ICM patients (P=0.001 and <0.001 for loop diuretics and spironolactone, respectively). Sacubitril/Valsartan was initiated and tolerated more frequently among DCM patients than ICM patients (177, 46% VS 145, 22%; P>0.001). Moreover, in patients who were treated with Sacubitril/Valsartan, the dose could be titrated higher during the short-term follow-up period among DCM patients than among ICM patients (57% VS 29%; P<0.001). Conversely, ACEIs/ARBs were utilized more frequently among ICM patients than DCM patients (65% VS 45%; P<0.001). With regard to the prevention of sudden cardiac death and improving both quality of life as well as mortality, the utilization of device therapy (ICDs/CRTDs) was observed to be significantly higher among DCM patients than ICM patients (21% VS 11%; P=0.001), as shown in Table 3.

**Table 3:** Anti-failure treatment for both dilated and ischemic cardiomyopathy patients. ACEI: angiotensin-converting enzyme inhibitor; ARBs: angiotensin-receptor blockers; BB: beta-blockers; CRTD: cardiac resynchronization therapy device; ICD: implantable cardioverter-defibrillators; NS: not significant.

Variable	Dilated cardiomyopathy (DCM); N = 385 (36%)	Ischemic cardiomyopathy (ICM); N = 663 (62%)	P-value
Loop diuretics	323 (84%)	503 (76%)	0.001
Spironolactone	327 (85%)	424 (64%)	<0.001
Metolazone	23 (6%)	33 (5%)	NS
BB	355 (92%)	603 (91%)	NS
Digoxin	31 (8%)	13 (2%)	0.01
Sacubitril/Valsartan (Entresto)	177(46%)	145 (22%)	<0.001
Titration of Entresto doses	219 (57%)	192 (29%)	<0.001
ACEi/ARBs	173 (45%)	431 (65%)	<0.001
Device therapy (ICD/CRTD)	81 (21%)	73 (11%)	0.001

**Figure 3:**
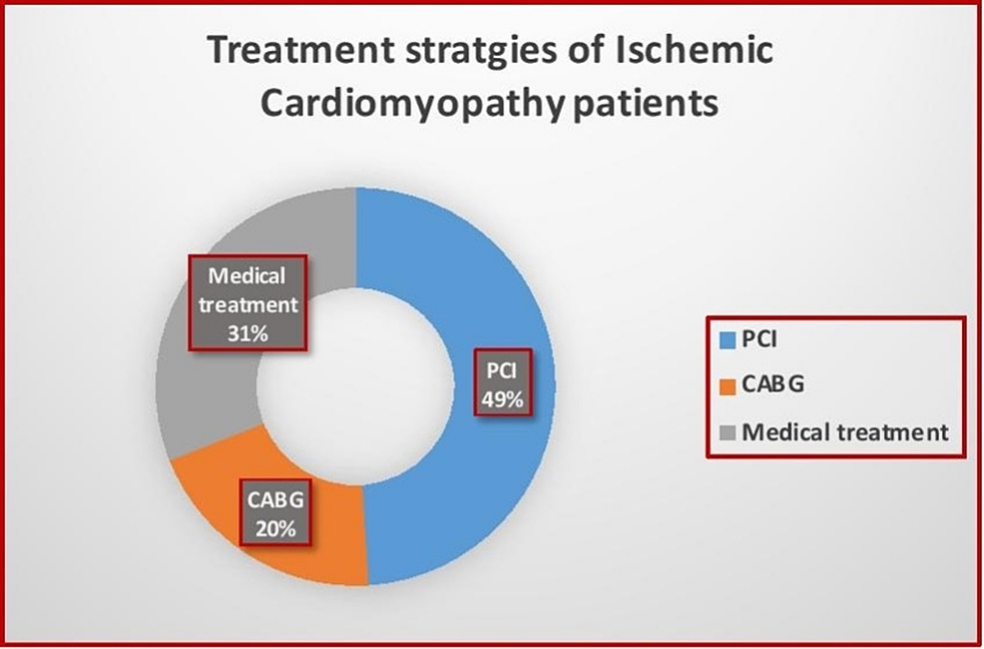
Treatment strategies selected for patients with ischemic cardiomyopathy. CABG: coronary artery bypass grafting; PCI: percutaneous coronary intervention.

Moreover, in addition to standard treatment for ICM patients, revascularization strategies were utilized more frequently than conventional medical therapy (49% for PCI, 20% for CABG VS only 31% for medical treatment), as shown in Figure 3.

In-hospital outcome measures and mortality 

ICM patients showed a higher prevalence of indication for mechanical ventilation during their hospital stay (P=0.05); however, DCM patients showed a higher rate of arrhythmias (P=0.001). The length of hospital stay did not differ between the two groups, nor did the prevalence of pulmonary edema, cardiogenic shock, or cardiac arrest in severe heart failure cases. The total in-hospital mortality was 16%, with a higher incidence among ICM patients than among DCM patients (19% VS 13%; P = 0.001), as shown in Table 4.

**Table 4:** Hospital outcome measures for both dilated and ischemic cardiomyopathy patients. NS: not significant.

Variable	Dilated cardiomyopathy (DCM); N = 385(36%)	Ischemic cardiomyopathy (ICM); N = 663(62%)	P-value
Death	50 (13%)	126 (19%)	0.001
Mechanical ventilation	9 (2%)	53 (8%)	0.001
Arrhythmias	116 (30%)	113 (17%)	0.05
Pulmonary edema	23 (6%)	60 (9%)	NS
Cardiogenic shock	15 (4%)	40 (6%)	NS
Cardiac arrest	20 (5%)	47 (7%)	NS
Length of hospital stay (days)	11.16± 7.2	12. 15± 8.6	NS

According to the multivariate analysis, independent predictors of mortality among cardiomyopathy patients were age, renal impairment, and lower LVEF (P= 0.04, <0.001, and 0.21, respectively), as shown in Table 5.

**Table 5:** Multivariate analysis of the predictors of mortality. CI: 95% confidence interval; CKD: chronic kidney disease; DM: diabetes mellitus; HTN: hypertension; LVEF: left ventricular ejection fraction; RR: relative risk.

Variable	RR (CI 95%)	P-value
Age	1.002 (1.000 –1.004)	0.004
Male gender	1.074 (0.97 – 1.18)	0.14
Pilgrims	1.051 (0.96 – 1.15)	0.29
DM	0.999 (0.99 – 1.003)	0.82
HTN	1.022 (0.95 – 1.10)	0.55
CKD	1.22 (1.12 – 1.33)	<0.001
Serum sodium	0.996 (0.990 –1.002)	0.23
LVEF%	1.081 (1.01 – 1.16)	0.02

## Discussion

What is already known about this subject, and what does this study add? 

Our center is the only cardiac center in the Mecca region providing tertiary care facilities including revascularization and advanced heart failure treatment. Because of this, it receives most of the cardiomyopathy patients deemed suitable for invasive assessment and advanced management. This led to a unique comparison between DCM and ICM patients regarding the severity, management, and outcome data. 

The results of our study have shown that ICM patients were older than patients with DCM which can be explained by coronary artery disease mainly affecting a more elderly age group [11]. Atherosclerosis-inducing coronary artery disease (CAD) is the most common cause of ischemic cardiomyopathy in old age and is characterized by a decreased blood supply that carries oxygen and essential nutrients to cardiac muscles; this leads to deterioration of the cardiac muscle function and chamber remodeling or dilation, which eventually lead to congestive heart failure (CHF) [11,12], Moreover, ICM is determined by several risk factors that include diabetes mellitus, hypertension, and renal impairment, as statistically proven in our study. 

Interestingly, we found that DCM patients showed a more severe presentation and had a higher prevalence of arrhythmias, making them more frequent candidates for device therapy than ICM patients. This might be explained by the fact that the DCM phenotype is mainly characterized by left ventricular dilatation and contractile dysfunction in the absence of hypertensive, valvular, congenital heart disease, or significant CAD [13]; however, the progressive dilatation can lead to weakness in the heart muscle, which further lowers the ejection fraction and increases the stress on the ventricular wall. Once symptoms develop, DCM usually leads to decompensated heart failure, and it represents one of the most common causes of heart transplantation in the Western world. Often life-threatening arrhythmias and sudden cardiac death (SCD) can characterize the course of DCM or represent the abrupt onset of the disease [14]. 

Proper diagnosis and further workup of admitted cardiomyopathy patients are crucial for the management and implementation of advanced therapy. Concerning echocardiography, our study showed many disparities between ICM and DCM patients, such as LV dilatation and systolic dysfunction, which were found to be poorer among DCM patients than ICM patients; this was consistent with other studies [15]. Furthermore, in a study that aimed to differentiate between ischemic and non-ischemic cardiomyopathy patient markers, RV enlargement was one of the differential echocardiogram findings in non-ischemic dilated cardiomyopathy patients [16]. This was also similar to our findings. 

The guideline-directed medical treatment recommended for all patients with decompensated heart failure includes diuretics, beta-blockers, renin-angiotensin system inhibitors (ACE inhibitor/ARB), and mineralocorticoid-receptor antagonists. Although Sacubitril/Valsartan is FDA approved for use in NYHA functional class II to IV patients with heart failure with reduced Ejection Fraction (HFrEF), data and guidance regarding its use are still limited [17]. Recently, a LIFE trial was designed to increase the amount of data regarding the safety and efficacy of Sacubitril/Valsartan in HFrEF patients. It also provided important information regarding its use in the management of patients with advanced HF [18]. This guided us in our study of the implementation of advanced therapy and its practical use among different heart failure patient populations. In our findings, DCM patients showed a higher need for advanced therapy, including Sacubitril/Valsartan. This is explained by the more severe symptoms that they presented with, and the lower recorded left ventricular ejection fractions noted in their echocardiograms compared to ischemic patients. Overall, the usage of higher doses of Sacubitril/Valsartan was the main goal in a tertiary care center follow-up of heart failure patients; this was limited by many factors such as side effects and noncompliance of the patients. The implementation of appropriate patient and clinician support pathways guides better uptake, dose-titration, and maintenance of evidence-based doses in clinical practice [19]. Our findings suggest that patients with DCM tolerate Sacubitril/Valsartan well, as evidenced by higher rates of dose titration compared to ICM patients. This can be explained by DCM patients being at a younger age with less associated morbidities, whereas ICM patients were elderly with multiple morbidities and had a higher prevalence of renal impairment, which limited drug use and dose titration among them. The long-term benefits of Sacubitril/Valsartan in the improvement of left ventricular ejection fraction among different types of cardiomyopathy patients have been explored in some recent trials and studies [20,21]. The long-term efficacy and safety of Sacubitril/Valsartan are not covered in the current study and will be a future topic of investigation. 

In terms of device therapy, a retrospective cohort study that included 153 consecutive patients (48 non-ischemic cardiomyopathy, 105 ischemic cardiomyopathy) reported that non-ischemic patients received more device therapy than ischemic patients [22]. This finding agrees with ours, which suggested that device implantation is used more frequently among DCM patients than ICM patients. This again might be explained by the fact that severe heart failure presentation, lower recorded LVEF, and life-threatening arrhythmias were all reported mainly among DCM patients [14]. 

Patients with ischemic cardiomyopathy may benefit from revascularization. There was a 7% absolute reduction in overall mortality over a 10-year time between patients who had CABG versus standard medical treatment [23]. Another meta-analysis of 21 studies comparing medical therapy using PCI and CABG in patients with heart failure revealed the paucity of revascularization therapy in ischemic cardiomyopathy with a significant reduction in mortality, and this is independent of viability testing [24]. We believe that the rate of revascularization therapy in our current study among ICM patients was appropriate and followed the standard strategies. These findings highlight the importance of the appropriate utilization of tertiary services in qualified cardiac centers and adherence to treatment guidelines. 

Overall, the prognosis of patients with cardiomyopathy depends on their disease state and chronicity. In ICM patients, a major component in the determination of their prognosis is myocardial viability and the use of revascularization therapy [11]. In DCM scenarios, most patients eventually end up with chronic heart failure and become candidates for advanced therapy [25]. In a recent study [26], the ICM group showed higher mortality rates and were more likely to have in-hospital complications compared to the DCM group, which is consistent with our findings. This might be explained by ICM patients being more elderly, having multiple morbidities, and presenting fewer candidates for advanced heart failure therapy. However, DCM patients were younger, presenting more candidates for the advanced treatment and might have potential reversibility of their disease. In-hospital mortality was independently predicted by age, renal impairment, and lower LVEF, which is consistent with other studies in the literature [27-29]. This also highlights that although we had patients with different characteristics, we found that most of the predictors of in-hospital mortality in our sample were very similar to those previously published in other studies. 

The results of our study should help to give the cardiovascular research community a deeper view of the prevalence, clinical manifestations, and severity of each type of cardiomyopathy, as well as how new approaches are most effectively used and allocated to the candidate patients. Our research also highlights the importance of increased awareness, implementation, and appropriate utilization of the advanced heart failure management and revascularization strategies that are available in tertiary centers. 

Limitations 

The present study had some limitations. Firstly, the number of enrolled patients was related to the nature of single-center and limited selection period, in addition to the situation of the COVID-19 pandemic. Secondly, selection bias cannot be excluded. Thirdly, no long-term follow-up data was collected due to the nature of tertiary care centers; most patients completed their follow-up in their primary and secondary hospitals. Finally, the number of non-ICM patients was small

Recommendations

Studies of larger numbers of patients and/or multicenter studies are needed to confirm the results of the present study. 

## Conclusions

There are discrepancies in the prevalence, demographics, clinical characteristics, and outcomes between dilated and ischemic cardiomyopathy patients. Although DCM patients were younger and had fewer cardiovascular risk factors, they presented with more severe symptoms and dysfunction, making them more likely candidates for advanced heart failure treatment, and they showed better outcomes reflected by a lower mortality rate. Increased age, presence of renal dysfunction, and lower LVEF were found as independent predictors of in-hospital mortality. 
